# Particulate matter (PM) 2.5 levels in ETS emissions of a Marlboro Red cigarette in comparison to the 3R4F reference cigarette under open- and closed-door condition

**DOI:** 10.1186/1745-6673-7-14

**Published:** 2012-06-26

**Authors:** Daniel Mueller, Johannes Schulze, Hanns Ackermann, Doris Klingelhoefer, Stefanie Uibel, David A Groneberg

**Affiliations:** 1 Institute of Occupational, Social and Environmental Medicine, Goethe-University, Frankfurt am Main, Germany; 2 Office of the Dean, Goethe-University, Frankfurt am Main, Germany; 3 Institute of Biostatistics and Mathematical Modeling, Goethe-University, Frankfurt am Main, Germany

## Abstract

**Introduction:**

Potential health damage by environmental emission of tobacco smoke (environmental tobacco smoke, ETS) has been demonstrated convincingly in numerous studies. People, especially children, are still exposed to ETS in the small space of private cars. Although major amounts of toxic compounds from ETS are likely transported into the distal lung via particulate matter (PM), few studies have quantified the amount of PM in ETS.

**Study aim:**

The aim of this study was to determine the ETS-dependent concentration of PM from both a 3R4F reference cigarette (RC) as well as a Marlboro Red brand cigarette (MRC) in a small enclosed space under different conditions of ventilation to model car exposure.

**Method:**

In order to create ETS reproducibly, an emitter (ETSE) was constructed and mounted on to an outdoor telephone booth with an inner volume of 1.75 m^3^. Cigarettes were smoked under open- and closed-door condition to imitate different ventilation scenarios. PM_2.5_ concentration was quantified by a laser aerosol spectrometer (Grimm; Model 1.109), and data were adjusted for baseline values. Simultaneously indoor and outdoor climate parameters were recorded. The time of smoking was divided into the ETS generation phase (subset “emission”) and a declining phase of PM concentration (subset “elimination”); measurement was terminated after 10 min. For all three time periods the average concentration of PM_2.5_ (C_mean_-PM_2.5_) and the area under the PM_2.5_ concentration curve (AUC-PM_2.5_) was calculated. The maximum concentration (C_max_-PM_2.5_) was taken from the total interval.

**Results:**

For both cigarette types open-door ventilation reduced the AUC-PM_2.5_ (RC: from 59 400 ± 14 600 to 5 550 ± 3 900 μg*sec/m^3^; MRC: from 86 500 ± 32 000 to 7 300 ± 2 400 μg*sec/m^3^; p < 0.001) and C_mean_-PM_2.5_ (RC: from 600 ± 150 to 56 ± 40 μg/m^3^, MRC from 870 ± 320 to 75 ± 25 μg/m^3^; p < 0.001) by about 90%. C_max_-PM_2.5_ was reduced by about 80% (RC: from 1 050 ± 230 to 185 ± 125 μg/m^3^; MRC: from 1 560 ±500 μg/m^3^ to 250 ± 85 μg/m^3^; p < 0.001). In the subset “emission” we identified a 78% decrease in AUC-PM_2.5_ (RC: from 18 600 ± 4 600 to 4 000 ± 2 600 μg*sec/m^3^; MRC: from 26 600 ± 7 200 to 5 800 ± 1 700 μg*sec/m^3^; p < 0.001) and C_mean_-PM_2.5_ (RC: from 430 ± 108 to 93 ± 60 μg/m^3^; MRC: from 620 ± 170 to 134 ± 40 μg/m^3^; p < 0.001). In the subset “elimination” we found a reduction of about 96–98% for AUC-PM_2.5_ (RC: from 40 800 ± 11 100 to 1 500 ± 1 700 μg*sec/m^3^; MRC: from 58 500 ± 25 200 to 1 400 ± 800 μg*sec/m^3^; p < 0.001) and C_mean_-PM_2.5_ (RC: from 730 ± 200 to 27 ± 29 μg/m^3^; MRC: from 1 000 ± 450 to 26 ± 15 μg/m^3^; p < 0.001). Throughout the total interval C_max_-PM_2.5_ of MRC was about 50% higher (1 550 ± 500 μg/m^3^) compared to RC (1 050 ± 230 μg/m^3^; p < 0.05). For the subset “emission” - but not for the other periods - AUC-PM_2.5_ for MRC was 43% higher (MRC: 26 600 ± 7 200 μg*sec/m^3^; RC: 18 600 ± 4 600 μg*sec/m^3^; p < 0.05) and 44% higher for C_mean_-PM_2.5_ (MRC: 620 ± 170 μg/m^3^; RC: 430 ± 108 μg/m^3^; p < 0.05).

**Conclusion:**

This method allows reliable quantification of PM_2.5_-ETS exposure under various conditions, and may be useful for ETS risk assessment in realistic exposure situations. The findings demonstrate that open-door condition does not completely remove ETS from a defined indoor space of 1.75 m^3^. Because there is no safe level of ETS exposure ventilation is not adequate enough to prevent ETS exposure in confined spaces, e.g. private cars. Additionally, differences in the characteristics of cigarettes affect the amount of ETS particle emission and need to be clarified by ongoing investigations.

## Introduction

Air pollution is hazardous to human health [1]. In industrialized countries people spend much more time indoors than outdoors, therefore indoor air pollution is highly relevant in these countries [2,3]. Tobacco smoke emissions of smoker, referred as environmental tobacco smoke (ETS), is an important contributor to indoor air pollution [4,5]. In some countries restrictive legislation has decreased ETS exposure from passive smoking in workplaces and public places [6]; however, ETS exposure in private homes or cars continues. Over 90% of the world’s population is still exposed to ETS in public places and at work, mainly due to incomplete smoke-free public health regulations [6,7].

Tobacco combustion forms more than 5 000 chemicals, many of which are toxic and known carcinogens [8,9]. Smoke exhaled after a puff is referred to as mainstream smoke (MS). Between puffs sidestream smoke (SS) is emitting from the smouldering tobacco product. It has the same chemical mixture but differs in the quantity of some substances. ETS is composed both from MS and SS of cigarettes.

Passive smokers, exposed to the combination of SS and exhaled MS, have been shown to suffer from premature mortality and increased morbidity. Based on population studies, Öberg et al. [6] estimated that annually 603 000 excess deaths are caused worldwide due to ETS exposure (1% of all global deaths). Increased attributable risks are calculated for diseases like sudden infant death syndrome (SIDS), cardiac illnesses and bronchial carcinoma [6,7,10]. In addition, acute or chronic exposure of children to ETS is linked with illnesses of the lower respiratory tract, persistent otitis media, aggravation of asthma and reduced lung function [7].

ETS particles are in the size range of the particulate matter fractions (PM fraction) PM_10_ and PM_2.5_[11]. Therefore these PM fractions can be used as marker compounds for ETS emission and uptake. PM_10_ particles are respirable and can reach the alveolar respiratory tract. PM_2.5_ particles are characterized by their long airborne retention time and the inability to be cleared efficiently by the human respiratory tract [12]. The vast majority of the chemicals found in ETS is assigned to the particulate phase, including nicotine and many carcinogenic substances, e.g. polycyclic aromatic hydrocarbons (PAHs) and tobacco-specific N-nitrosamines (TSNAs) [13,14]. Although PM_10_ and PM_2.5_ particles are transporting a great amount of toxic compounds into the peripheral respiratory system, only few studies have quantified indoor ETS exposure by PM measurement. In the line with the Tobacco Smoke and Indoor Air Quality Study (ToPIQS) we therefore quantified the exposure to ETS particulates of the PM_2.5_ fraction inside a small model space. The ETS was generated with a specifically designed and built, manually operated device (ETSE). PM_2.5_-particle concentration in the ETS from the 3R4F reference cigarette (RC) and the brand cigarette Marlboro Red (MRC) was measured in a telephone booth under open- and closed-door conditions.

## Material and methods

### Equipment

The ToPIQ analysis platform included a manually operating Environmental Tobacco Smoke Emitter (ETSE) mounted on a side panel of a telephone booth. PM_2.5_ concentration, temperature, relative humidity, wind velocity and atmospheric pressure were recorded within the booth by a measuring system. Temperature and relative humidity were additionally recorded outdoors.

### Environmental tobacco smoke emitter

In order to create ETS from cigarettes in a realistic pattern an ETSE was designed and built. It consisted of a bag valve mask (BVM) connected by rubber tubes to a cigarette holder with a burning cigarette inside. By passive inflation and manual compression of the bag the ETSE simulated the smoking pattern of a human smoker. Due to negative pressure on the cigarette during inflation, mainstream smoke (MS) was transported from the cigarette into the bag and was subsequently vented into the telephone booth through manual pressure on the bag. Together with the sidestream smoke (SS) emitted by the smoldering cigarette, both smoke emissions formed the ETS inside the telephone booth. Inflation and compression of the bag was performed according to a smoking protocol and synchronized by acoustic signals of a metronome.

### Smoking protocol

All cigarettes were smoked following the same smoking protocol. The parameters of the protocol, e.g. puff number and puff duration were determined by parameters of a smoke pattern analysis. Every cigarette of this study was smoked similarly with a number of 15 puffs, a puff duration of 3 sec and an inter-puff lag of 15 seconds.

### Telephone booth

ETS was created and measured inside a discarded telephone booth with an inner volume of 1.75 m^3^ similar to the inner volume of a small car.

In exchange for a window a plexiglass panel was installed to attach the ETSE to the telephone booth and to give an insulated access for the hands and the tubing into the inside of the booth. Cigarettes were ignited and put out through this access too.

### Measuring system

PM_2.5_ concentration was quantified in real-time by a laser-aerosol spectrometer (GRIMM Technologies, Inc., Model 1.109; Ainring) with a time resolution of 6 sec and a particle sensitivity between 0.25 and 32 μm. The spectrometer was an integrated component of a cased measurement unit that was positioned inside the telephone booth. With a PCE-MSR 145 S-THP data logger (PCE Instruments; Meschede), another part of the measurement unit, indoor temperature, relative humidity and atmospheric pressure were recorded additionally in real-time. A PCE 007 rotating cup anemometer (PCE Instruments; Meschede) was attached at the upper door gap measuring the indoor wind velocity in real-time. The outdoor relative humidity and temperature was quantified by a PCE-HT 71 N data logger (PCE Instruments; Meschede) mounted on the outside wall of the telephone booth.

### Data processing and analysis

Each measurement consisted of a 5 min baseline measurement, for the correction of baseline values, and a 10 min measurement (referred as “total interval”), which started with the ignition of the cigarette and ended with the cut of time. For the analysis of the ETS emission during and after the active generation, we divided the time of total interval into two subsets: the first subset “emission” (increase in PM- concentration) consisting of the first 4.3 minutes, and the subset “elimination” (concentration decrease) consisting of the following 5.7 minutes. The area under the PM_2.5_ concentration curve (AUC-PM_2.5_) and the arithmetic mean PM_2.5_ concentration (C_mean_-PM_2.5_) were calculated for the total interval and its two subsets, as well as the arithmetic mean of the climate data. The maximum concentration (C_max_-PM_2.5_) was determined over the total interval.

Due to the division of the measurements, all experiments were performed in triplicate at least; ETS exposure parameters (AUC-PM_2.5_, C_mean_-PM_2.5_, C_max_-PM_2.5_) and climate parameters were tested for significant differences between the cigarette types, as well as under open versus closed door conditions with the Mann–Whitney U-test; a significant difference was assumed at α < 0.05.

### Environmental tobacco smoke emitter design

Cigarettes were smoked according to a smoking protocol using parameters, e.g. puff length, puff interval and total smoking duration, of smoking pattern analyses in published literature and of standard protocols for smoking machines (e.g. FTC and ISO). The negative pressure during the puff is not adjustable with the manual ETS emitter (ETSE). Therefore, not all parameters could be implemented in the smoking protocol used. Initial smoking protocols with the ETSE used a 2 sec puff duration according to standard the FTC and ISO machine-smoking method [15]. Uthese conditions the cigarette was completely smoked (maximal 5 mm before filter tip) after 18 puffs, not consistent with observed parameters of the smoking pattern analyses (about 8 – 16 puffs/cigarette; [16]). To imitate the identified smoke pattern the puff duration was increased to 3 sec. By this modification cigarettes were smoked within 15 puffs and thus within the range observed in real smokers.

## Results

### Open-door versus closed-door condition

**Figure 1:**
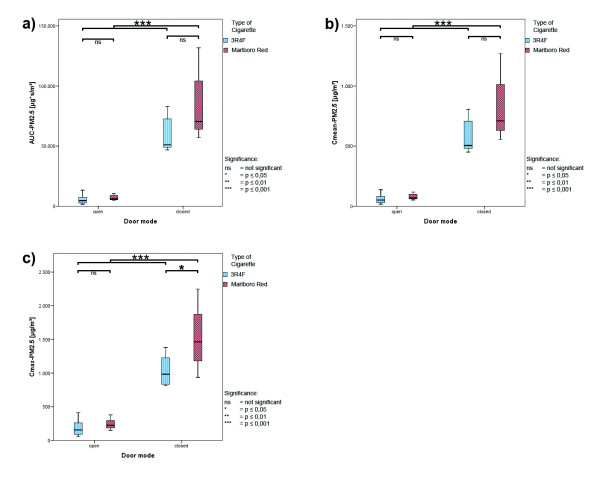
**Boxplots of ETS exposure; total measurement interval. a**) AUC-PM_2.5_; **b**) C_mean_-PM_2.5_; **c**) C_max_-PM_2.5_-concentration for 3R4F and Marlboro Red cigarettes;data were analyzed under open- and closed-door conditions.

In order to imitate ventilation, the telephone booth was used with a closed-door and an open-door mode. The open-door mode resulted in a massive decrease of PM_2.5_ parameters (appr. 90% decrease for AUC and C_mean;_ appr. 80% decrease for C_max_) for both cigarettes (p ≤ 0.001, all parameters), as expected. Box whisker plots of the ETS-generated PM_2.5_ concentration (AUC, C_mean_ and C_max_) are presented in Figure 1.

The AUC for PM_2.5_ decreased after door-opening by 91% (RC: from 59 400 ± 14 600 to 5 550 ± 3 900 μg*sec/m^3^; MRC: from 86 500 ± 32 000 to 7 300 ± 2 400 μg*sec/m^3^). This was mirrored by a similar reduction in C_mean_-PM_2.5_ also by 90% (RC: from 600 ± 150 to 56 ± 40 μg/m^3^, MRC from 870 ± 320 to 75 ± 25 μg/m^3^). The maximum concentration was 83% lower and was decreased for RC from 1 050 ± 230 to 185 ± 125 μg/m^3^, for MRC from 1 560 ± 500 μg/m^3^ to 250 ± 85 μg/m^3^.

**Figure 2:**
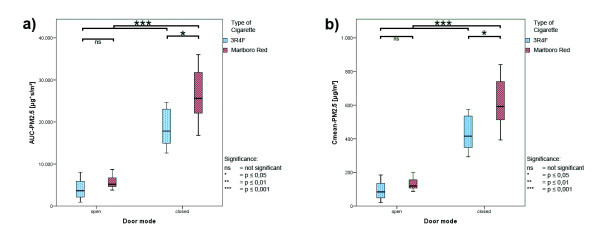
**Boxplots of ETS exposure; emission period. a**) AUC-PM_2.5_; **b**) C_mean_-PM_2.5_ for 3R4F and Marlboro Red cigarettes; data were analyzed under open- and closed-door conditions.

**Figure 3:**
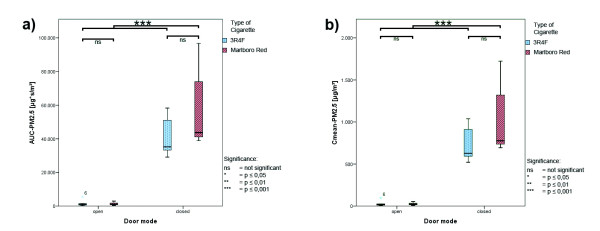
**Boxplots of ETS exposure; elimination period. a**) AUC-PM_2.5_; **b**) C_mean_-PM_2.5_ for 3R4F and Marlboro Red cigarettes; data were analyzed under open- and closed-door conditions.

The changes observed for the total interval were also seen in both subsets with a significant drop of the PM_2.5_ parameters under open-door condition (p ≤ 0.001 for all parameters), the magnitude, however, differed between the two periods. Box whisker plots of the AUC-PM_2.5_ and C_mean_-PM_2.5_ concentrations for both cigarette types under open- and closed-door conditions are presented in Figure 2 (emission period) and Figure 3 (elimination period). 

In the emission period the AUC-PM_2.5_ decreased under open-door conditions for both cigarettes by about 78% (RC: from 18 600 ± 4 600 to 4 000 ± 2 600 μg*sec/m^3^; MRC: from 26 600 ± 7 200 to 5 800 ± 1 700 μg*sec/m^3^) and the C_mean_-PM_2.5_ by about 79% (RC: from 430 ± 108 to 93 ± 60 μg/m^3^; MRC: from 620 ± 170 to 134 ± 40 μg/m^3^). In the elimination period the AUC-PM_2.5_ was reduced under open-door condition for both cigarettes by about 97% (RC: from 40 800 ± 11 100 to 1 500 ± 1 700 μg*sec/m^3^; MRC: from 58 500 ± 25 200 to 1 400 ± 800 μg*sec/m^3^) and the C_mean_-PM_2.5_ by about 96–97% (RC: from 730 ± 200 to 27 ± 29 μg/m^3^; MRC: from 1 000 ± 450 to 26 ± 15 μg/m^3^).

#### Reference cigarette versus brand cigarette

When comparing the emission kinetic parameters for the cigarette types significant differences were found only under closed-door conditions although the relative difference was comparable for all parameters (cf. Figure 1 to Figure 3).

For the total interval only differences in the C_max_-PM_2.5_ (p < 0.05) for Marlboro Red cigarettes being 50% higher than the values of the 3R4F reference cigarette (MRC: 1 550 ± 500 μg/m^3^ and RC: 1 050 ± 230 μg/m^3^;) reached significance (p < 0.05). Other parameters (AUC-PM_2.5_ and C_mean_-PM_2.5_) were higher in the ETS of Marlboro Red cigarettes; however, the difference did not reach significance.

During the emission period significantly higher AUC-PM_2.5_ and C_mean_-PM_2.5_ (approx. 43% for both parameters) were measured in the ETS of Marlboro Red cigarettes (p < 0.05). Whereas for Marlboro Red cigarettes an AUC-PM_2.5_ of 26 600 ± 7 200 μg*sec/m^3^ and a C_mean_-PM_2.5_ of 620 ± 170 μg/m^3^ was calculated under closed-door condition, the corresponding values for 3R4F reference cigarettes were 18 600 ± 4 600 μg*sec/m^3^ (AUC-PM_2.5_) and 430 ± 108 μg/m^3^ (C_mean_-PM_2.5_).

For the elimination period differences between the ETS-PM_2.5_ parameters (AUC-PM_2.5_ and C_mean_-PM_2.5_) were noticeable as seen in Figure 3, but did not reach significance.

### Climate parameters

**Figure 4:**
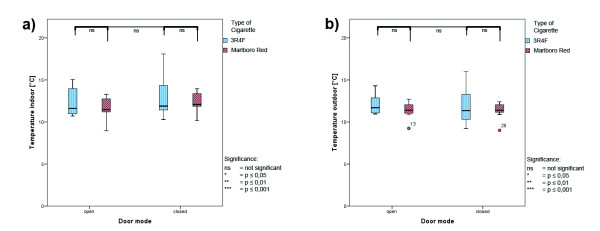
**Climate parameters during ETS measurement - temperature. a**) indoor temperature; **b**) outdoor temperature; red: Marlboro Red; blue: 3R4F reference cigarettes; values are averages for the total time interval.

**Figure 5:**
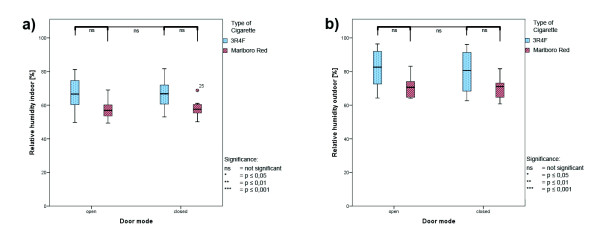
**Climate parameters during ETS measurement – relative humidity. a**) indoor relative humidity; **b**) outdoor relative humidity; red: Marlboro Red; blue: 3R4F reference cigarettes; values are averages for the total time interval.

**Figure 6:**
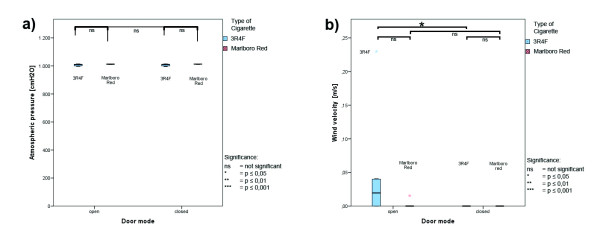
**Climate parameters during ETS measurement – air pressure and wind velocity. a**) atmospheric pressure; **b**) wind velocity; red: Marlboro Red; blue: 3R4F reference cigarettes; values are averages for the total time interval.

Climate parameters were collected simultaneously throughout the total measurement interval; box whisker plots are presented in Figures 4, 5 and 6. No differences between open-door and close-door conditions were seen during the measurements, except significant higher values for wind velocity during open-door conditions. Higher wind velocity due to door opening can be expected. The uneven distributed wind velocity values between the cigarette types reflecting the variable wind conditions in open-door measurements. Table 1 lists the absolute values for all climate parameters. 

**Table 1:** Climate parameters

	**Relative Humidity**		**Temperature**		**Atmospheric Pressure**	**Wind velocity**
	**[%]**		**[°C]**		**[cmH_2_O]**	**[cm/s]**
	**Mean ± SD**		**Mean ± SD**		**Mean ± SD**	**Mean ± SD**
	**indoor**	**outdoor**	**indoor**	**outdoor**	**indoor**	**indoor**
**open-door measurement**						
3R4F	67 ± 11	82 ± 12	12 ± 2	12 ± 1	1.007 ± 7	5,6 ± 7,9
Marlboro Red	57 ± 6	71 ± 7	12 ± 1	11 ± 1	1.012 ± 1	0,75 ± 1,5
**closed-door measurement**						
3R4F	67 ± 9	80 ± 13	13 ± 3	12 ± 2	1.007 ± 7	< 0,001
Marlboro Red	58 ± 6	70 ± 7	12 ± 1	11 ± 1	1.013 ± 1	< 0,001

Absolute values for climate parameters (mean and standard deviation) are recorded during the ETS measurements of 3R4F reference and Marlboro Red cigarettes under open-door and closed-door conditions.

## Discussion

In previous studies that measured indoor PM concentration of ETS, the smoke was either generated by human smokers [17-20] risking health impairment, or by smouldering cigarettes thus producing only SS with no MS [18,21,22]. The ETSE used in this study produced both MS as well as SS in a pattern similar to the behavior of real smokers, but without exposure to humans. The used puff duration of 3 sec was about 1 sec longer than the puff duration according to smoke pattern analyses (average 1.8 sec) or standard machine-smoking protocols [15,16]. This prolonged puff duration reduced the number of puffs from 18 to 15 as observed by smoking pattern analyses (8–16 puffs / cigarette) [16]. It can be assumed that the ETSE is not able to build up the same air flow rate on the cigarette during a puff as a human smoker. The flow rate of the ETSE depends on passive inflation of the bag by relaxation and is therefore not adjustable. Thus, ETS generated by the ETSE may differ from ETS emitted by a smoker. In order to quantify the effects of a longer puff duration as opposed to increased flow rate, further comparisons may be necessary. Furthermore, the ETSE does not simulate the deposition of smoke in a smoker’s lung before exhaling the MS. The estimated proportion of smoke deposition varies greatly [11]. Comparative studies measuring the PM concentration in emitted ETS by smokers and by the ETSE could clarify the differences in smoke deposition between the human lung and ETSE.

In order to quantify the ETS exposure the ETS-dependent PM_2.5_ concentration was measured. Morawska et al. have proposed PM_2.5_ as a suitable marker for the submicron part of ETS, whose size is bimodally distributed [11]. In the submicron part of ETS they found a greater number of particles with less than 1 μm in diameter compared to the supermicron part (particles >1 μm) that contains a larger fraction of the total ETS mass [11].

Like other studies on ETS particulates, the arithmetic mean (C_mean_-PM_2.5_) and the maximum concentration (C_max_-PM_2.5_) of PM_2.5_-ETS has been calculated. These parameters indicate the average and peak exposure to exposed individuals and are used in air quality guidelines and most publications.

To the best of our knowledge, the area under the curve (AUC) – a parameter commonly used in pharmacokinetics and toxicokinetics – has not been used yet in ETS research studies. This parameter allows the quantification of a level of burden attributable to a specific source, e.g. a cigarette; it may be used to compare different sources for toxicants, which are emitted discontinuously. For continuous exposure the AUC is simply calculated as the concentration-time product for a given period.

AUC of the PM_2.5_ concentration (AUC-PM_2.5_) was used as a comparable parameter for the exposure to ETS particulates. If Haber’s rule (the product of concentration (c) and time of exposure (t) is constant (k); c × t = k) can be applied the total biological response, e.g. cancer [23], will be identical to the sum of all exposures. AUC values for cigarette ETS allows to compare different exposure scenarios. Rozman concluded that Haber’s rule can also be applied if the toxicant reaches a toxicokinetic steady state and/or the biologic effect of the toxicant is irreversible [24]. Some of the ETS toxicants may meet these conditions too, e.g. carcinogenic substances. According to the WHO Air Quality Guidelines (2000) the integral of a concentration over a long period can have more impact on health than the pattern of peak exposure [25].

A telephone booth (type: TelH 78) was chosen as a model for a confined indoor space like a car. Because of its simple shape and the small inner volume of 1.75 m^3^ the telephone booth is suitable for a fast homogenization of ETS particles with the indoor air. This mixing process was supported by the repetitive compressions of the ETS bag during ETS generation. Natural ventilation was given through door slits and perforations in the ceiling of the telephone booth and its effect was documented by a rotating cup anemometer. The size of the telephone booth is comparable to the size of a small car. By door opening an open-windows condition of a parked car can be simulated with the booth.

Using our smoke emitter and enclosed space, our data show that indoor measurement result in realistic data. The Effect of door opening reduced ETS-PM_2.5_ by 80–90% as should be expected. However, even with this large reduction a C_mean_-PM_2.5_ of about 56 ± 40 μg/m^3^ (RC) and 75 ± 25 μg/m^3^ (MRC) was found and indicates a considerable exposure. Since there is no safe ETS level [5] ventilation cannot prevent harmful ETS exposure in spaces comparable to a telephone booth, e.g. inside small private cars. A major ETS exposure can be expected in larger cars with a larger inner volume as well (the passenger cabin volume of large class car is about 120 ft^3^ = 3 m^3^ according to 40C.F.R § 600. 315–82 (1982)).

Our findings also show that compared to other parameters of PM concentration the maximal exposure concentration (C_max_-PM_2.5_) is reduced less by ventilation, independent of cigarette type. Thus, for acute toxic compounds in tobacco smoke like acrolein it can be expected that the ventilation effect is even lower than the decrease in carcinogens.

Major differences were seen in the PM_2.5_-parameters of ETS emissions between the cigarette types investigated; they were significant only for some parameters. In comparison to the 3R4F reference cigarette 43% higher values for AUC-PM_2.5_ as well as for C_mean_-PM_2.5_ were found for the Marlboro Red cigarette, and about 50% higher values for C_max_-PM_2.5_. It can be assumed that one reason for the higher values is their shorter filter length. The filters of both cigarettes are similar in material (cellulose acetate and triacetin) and diameter [26,27]. However, the Marlboro Red cigarette filter is 6 mm shorter (RC: 27 mm; MRC: 21 mm; [26], confirmed by own measurement). Both cigarettes also differ by tobacco additives and their filter paper, which may influence the ETS particle concentration as well.

Few studies have addressed the effect of additives on particle release into tobacco smoke. Nevertheless, these studies investigated only MS and not ETS; their results are also inconsistent. Rustermeier et al. (2002) observed 13 – 28% increased total particulate matter (TPM) in MS of cigarettes with additives [28]. They assumed that a higher transfer rate of the added ingredient to the smoke could cause a higher TPM yield. Baker et al. on the other hand could not find a difference between the TPM yields in MS of cigarettes with or without additives [29].

In this paper a reliable method to quantify PM_2.5_ concentrations in ETS is provided; the method can be modified to simulate different environmental conditions. We calculated AUC-PM_2.5_ values to quantify the exposure with PM_2.5_ in ETS and compared the exposure on the basis of identifiable sources. We believe this toxicological parameter can be useful for comparing sources and for the risk assessment of different exposure situations.

Ventilation alone cannot abolish smoke related exposures even with an intensive ventilation pattern. With ventilation, peak exposure concentrations were decreased less than average concentrations or AUC values. Further research is necessary to elucidate whether this effect translates into specific damage for vulnerable people like children or pregnant women. Our method allows to compare ETS exposure with different smoking habits, ventilation scenarios, room sizes and cigarette types. It may help to put the health impact of smoking into perspective.

## Competing interests

The authors declare that they have no competing interests.

## Authors’ contributions

DM, JS, HA, DK, SU, DAG have made substantial contributions to the conception and design of the study, the acquisition of data and the involvement of drafting and revising the manuscript. All authors have read and approved the final manuscript.
